# Identification of smoking cessation phenotypes as a basis for individualized counseling: An explorative real-world cohort study

**DOI:** 10.18332/tid/152546

**Published:** 2022-09-23

**Authors:** Maciej Paciorkowski, Florent Baty, Susanne Pohle, Esther Bürki, Martin Brutsche

**Affiliations:** 1 Lung Center, Cantonal Hospital St. Gallen, St. Gallen, Switzerland

**Keywords:** smoking cessation, personas, principal coordinates analysis, clusters, individualized treatment

## Abstract

**INTRODUCTION:**

The rate of relapse in smokers attempting to quit is generally high. In order to maximize the chances of success, it is of interest to better understand the dynamic of lapse and relapse during smoking cessation. We hypothesized that specific behavioral patterns in tobacco consumption could predict the probability of quitting success and could open the possibility for a more targeted approach. The aim of the current study was to characterize clusters of quitting trajectories among participants involved in a smoking cessation program.

**METHODS:**

In a retrospective real-world cohort study, data from 843 consecutive participants between March 2012 and December 2014 were collected. Data consisted of baseline information on demographics, smoking history and dependence level, as well as longitudinal data about tobacco consumption. The correlations among time series were characterized using principal coordinates analysis. Clusters were identified using *k*-means clustering and the average profile associated with each cluster was computed. The association between the participant’s baseline characteristics and clusters of tobacco consumption was assessed.

**RESULTS:**

Four distinct clusters of transition phenotypes were identified based on tobacco consumption during the cessation phase: the long-term quitters (30%), the persistent smokers/reducers (44%), the short-term returners (16%) and the repeated try and failers (10%). Significant between-cluster differences were found in terms of baseline characteristics and smoking behavior during follow-up.

**CONCLUSIONS:**

Meaningful clusters of quitting trajectories could be identified. Such specific behavioral patterns were useful for the application of personalized assistance needed to achieve successful and long-term cessation.

## INTRODUCTION

Tobacco smoking is a risk factor for a number of chronic diseases, a leading cause of preventable deaths and as such a major public health problem^1,2^. Smokers who quit smoking can substantially reduce their risk of developing and dying from tobacco-related diseases^3,4^. Two-thirds of smokers declare that they are willing to quit and approximately half of them say that they tried to quit in the past year^5^. Individual smoking cessation counseling increases the probability of cessation in comparison to minimal contact control from 40% to 80% after 6 months^6,7^. Due to increased digitalization, many auto-didactic tools, including mobile applications and web-based programs, have been developed for supporting smokers during the quitting process^8,9^.

A number of smoking cessation trials suggest that quitting is a complex process that can occur either abruptly or gradually, or following a series of lapses and relapses. Only a small minority of smokers actually change in a linear manner without experiencing any relapse. Relapse may be seen as a part of the smoking cessation process. Therefore, it is critical to identify and resolve the source of relapse. After a failed quit attempt smokers should try again, because many of them require several attempts before achieving lasting abstinence^10,11^.

The characterization of the dynamic of lapse and relapse during smoking cessation may bring a better understanding on the factors predicting the chances of quitting success. Several attempts have been made for the identification of distinct quitting trajectories during smoking cessation^12-16^. These studies typically demonstrate the existence of 3 to 5 distinct quitting patterns (Table 1).

**Table 1:** Transition phenotypes during assisted smoking cessation as identified by former studies

*Authors Year*	*Sample size*	*Follow-up time (days)*	*Number and description of the phenotypes*
Bachmann et al.^12^ 2012	230	29	4: Quitters; Late quitters; Returners; Persistent smokers
Conklin et al.^13^ 2005	108	365	5: Abstinent; Low-level users; Moderate users; Slow-returners; Quick-returners
Wong et al.^14^ 2011	402	180	3: Reducers; Persistent smokers; Quitters
Hoeppner et al.^15^ 2008	57	40	3: Increasing; Constant; Decreasing
Cofta-Woerpel et al.^16^ 2011	300	28	3: Abstainers; Early lapsers; Late lapsers

The aim of the current study was to characterize the patterns of lapse and relapse in participants undergoing a smoking cessation intervention using an original distance-based exploratory statistical approach that allows visualizing similarities in the participant’s smoking behavior. We intended to identify clusters among the quitting trajectories and investigate their significance in terms of quitting success. This is one of the first studies demonstrating a potential role of phenotyping smokers attempting to quit smoking as well as an innovative use of ‘personas methodology’.

## METHODS

### Study population

**Figure 1:**
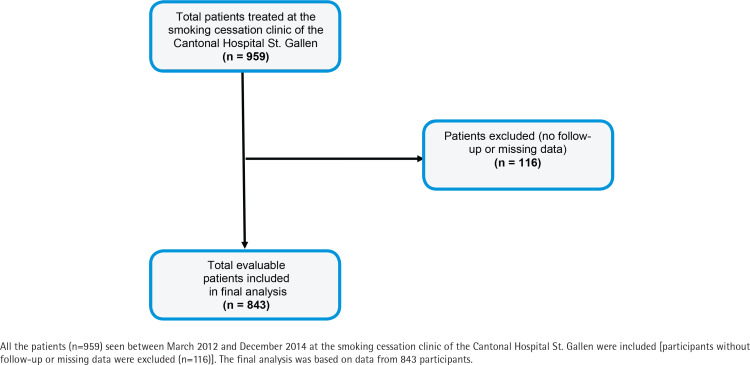
Study flow chart

The current study is based on single center, realworld, retrospective data from participants treated at the smoking cessation clinic of the Cantonal Hospital St. Gallen between March 2012 and December 2014. Most participants were referred by personnel of the Cantonal Hospital St. Gallen (mostly during a hospital stay because of comorbidities) and also by family physicians (on request of patients). Data of all consecutive participants (n=959) seen in our smoking cessation clinic were included. Participants without follow-up or missing data were excluded (n=116). The final analysis was based on data from 843 participants (Figure 1). The smoking cessation clinic at our hospital uses a combination of cognitive behavior therapy and pharmacotherapy, through multiple contacts with professional counselors. At the first visit, nicotine dependence using the Fagerström test score (low: 0–2, medium: 3–4, high: 5–10) and motivation level (pre-contemplative, awareness, preparation, active) have been regularly assessed by a counselor. Furthermore, regular self-assessment to quit on motivation and self-confidence scales (score range: 0–10) has been done. Follow-up visits were performed using face-to-face appointments and/or phone contacts. For the sake of the current analysis, the maximum follow-up time was restricted to 6 months. Data retrieved from our institutional patient information system were analyzed anonymously.

### Patient and public involvement

Patients or the public were not involved in the design, or conduct, or reporting, or dissemination plans of our research.

### Statistical considerations and cluster identification

Longitudinal data regarding the participants’ tobacco consumption were converted into irregular time-series objects. Irregular time series allow handling of data where the events are unevenly spaced in time, which applies to the current tobacco consumption data that consist of an irregular sequence of consumption data. The advantage of using irregular time series is that no imputation for missing values is required. The distances between each pair of individual time series were compiled into a distance matrix using the dynamic time warping distance. Time series were ordinated using principal coordinates analysis (PCoA), also known as multidimensional scaling, which is particularly adapted for the analysis and visualization of multivariate time series^17^. Clusters were identified using *k*-means clustering and the optimal number of clusters was estimated graphically using the Elbow method. The average profile (prototype) associated with each cluster was determined for visualization purposes. The interpretation of the clusters in the light of the dynamic of changes in lapse and relapse of tobacco consumption was provided using a vector fitting procedure^18^, which identifies the directions of maximal correlation with external variables in the PCoA space. All analyses were done using the R statistical software (v. 4.0.1)^19^ together with the extension packages *tseries*^20^, *zoo*^21^, *dtwclust*^22^ and *TSdist*^23^ for time-series analysis and *clustering*, as well as *ade4*^24^ and *vegan*^25^ for multivariate ordination and visualization.

### Personas methodology

The association between the participant’s baseline characteristics/outcomes and the identified clusters of tobacco consumption was further assessed. Participants’ profiles were further characterized using the personas methodology, which was first introduced in 1990s by Alan Cooper^26^. The personas approach is based on the construction of hypothetical user archetypes, which allow the better understanding of a client’s or patient’s needs, goals or behavior, and has been employed successfully in a variety of medical domains to clustering and providing support for patients^27-29^. In our study, the phenotypic personas descriptors were defined with the aid of consensus statements resulting from two inter-professional expert consensus workshops. The team in our smoking cessation clinic included trained counselors and pulmonologists. Expert consensus workshops took place and allowed discussion of the relevance of the smoking cessation clusters and defining the corresponding personas.

Personas have been developed on the basis of the findings from the cluster analysis as well as by consensus statements from inter-professional expert workshops. According to the findings of our study (phenotypes of patients) and our observations in the smoking cessation clinic, we created personas with specific distinctive features and stereotypes. The personas methodology was used as an attempt to describe the main features of the participants within each transition phenotype in order to help classify participants and simplify treatment modalities. A particular emphasis was placed on management-relevant characteristics, which in turn can be used to determinate the intervention model. The preferences of the patients corresponded to our observations of treatments administered in our smoking cessation clinic, as well as results from previously published studies regarding smoking cessation interventions and clinical guidelines for smoking cessation. The cartoon profile pictures were created using a free website service (www.cartoonify.de).

## RESULTS

### Participants’ characteristics

The baseline participant’s characteristics are summarized in Table 2. The median number of visits was 4 (IQR: 2–6) and the median follow-up time was 15 days (IQR: 1–84). Overall, 39% of participants had a high nicotine dependence and 38% were already in the active cessation process at the time of enrollment.

**Table 2:** Participants’ baseline characteristics of patients treated at the smoking cessation clinic of the Cantonal Hospital St. Gallen between March 2012 and December 2014 (N=843)

*Characteristics*	*n (%) or Median (IQR)*
**Males**	497 (59)
**Age** (years)	55 (45–62)
**Baseline tobacco consumption** (cigarettes/day)	20 (19–30)
**Pack-years**	40 (25–60)
**Age of smoking initiation** (years)	17 (15–20)
**FTND score** (0–10)	
Low (<2)	160 (19)
Moderate (3–4)	342 (41)
High (≥5)	325 (39)
**Motivation level**	
Pre-contemplative	8 (1)
Awareness	267 (32)
Preparation	248 (30)
Active	317 (38)
**Motivation scale** (0–10)	8 (5–9)
**Self-confidence** (0–10)	7 (5–8)

IQR: interquartile range. FTND: Fagerström test for nicotine dependence.

### Identification of smoking cessation phenotypes

**Figure 2:**
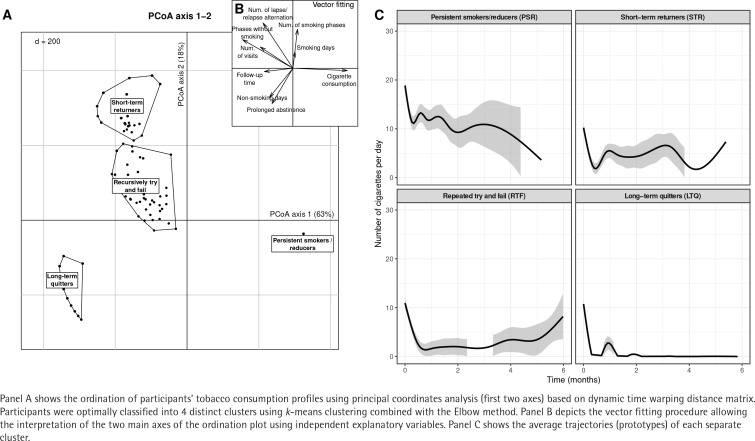
Cluster analysis with identification of smoking cessation phenotypes based on tobacco consumption during assisted smoking cessation

The results of the cluster analysis are shown in Figure 2. Four distinct clusters were identified using *k*-means clustering. The optimal number of clusters (k=4) was defined using the Elbow method (data not shown). The following four patterns were identified: long-term quitters (LTQ; n=252), persistent smokers/reducers (PSR; n=371), short-term returners (STR; n=135), and repeated tries and failures (RTF; n=85). A reliability of the four phenotypes identified by principal coordinates analysis using a bootstrap resampling strategy in order to visualize the confidence regions of PCoA solutions is shown in Supplementary file Figure 1.

### Characteristics of smoking cessation phenotypes

**Figure 3:**
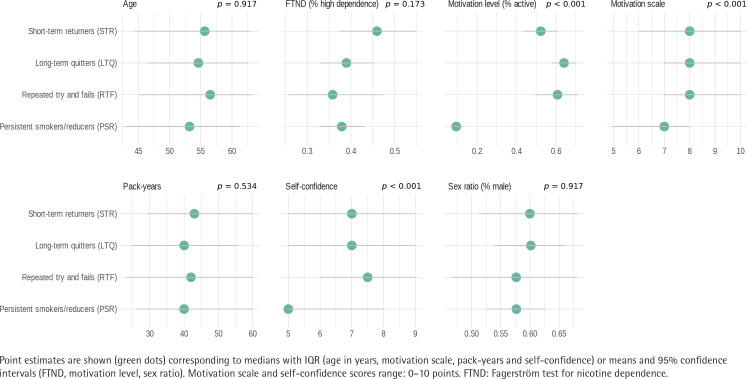
Baseline characteristics of the 4 transition phenotypes. The differences in baseline characteristic of participants are associated with the outcome and they are also clinically relevant as they allow placing particular emphasis on patient’s needs and possible challenges during smoking cessation

Baseline characteristics of the transition phenotypes of tobacco consumption are summarized in Figure 3. LTQ and RTF tended to be older than the PSR with a mean age difference of 2 years (95% CI: 0–4, p=0.045) and 3 years (95% CI: 0–6, p=0.055), respectively. There was a significant difference between the motivation levels of the 4 clusters (p<0.001). The proportion of participants in an active motivational phase was 64%, 61% and 52%, among the LTQ, RTF and STR, respectively, in comparison with only 9% among the PSR. No significant difference was found between the 4 clusters in terms of Fagerström test for nicotine dependence (p=0.173). On a motivation scale from 0 to 10, all participants among the LTQ, RTF and STR had a significantly higher motivation in comparison with the PSR, with a mean difference of 1.44 points (95% CI: 1.05–1.82, p<0.001), 1.44 points (95% CI: 0.86–2.02, p<0.001), and 1.38 points (95% CI: 0.89–1.87, p<0.001), respectively. Finally, a significantly higher self-confidence (measured on a scale from 0 to 10) was found among LTQ, RTF and STR in comparison with PSR, with a mean difference of 1.26 points (95% CI: 0.81–1.71, p<0.001), 1.24 points (95% CI: 0.59–1.9, p<0.001), and 0.99 points (95% CI: 0.46–1.53, p<0.001), respectively. The differences in baseline characteristics of participants of smoking cessation are associated with the outcomes and they are also clinically relevant as they allow placing particular emphasis on patient’s needs and possible challenges during a treatment.

### Outcome of smoking cessation

Overall, 251 out of 843 individuals (30%) reached a smoking-free state at 1 month. The outcome differed between the different transition phenotypes. Almost half of the participants were PSR, i.e. they never managed to quit smoking during the observation period. On the other hand, 30% of the patients were immediately successful (participants with constant abstinence from the first visit until the last follow-up). Participants who relapsed with intermittence belonged to the STR and RTF clusters representing 26% of all participants. LTQ were typically associated with longer phases of tobacco abstinence. They had a median time to first abstinence of 2 days (IQR: 2–5) and 94% of LTQ participants achieved prolonged abstinence within 30 days following their initial visit. RTF had longer follow-up time (median: 118 days; IQR: 43–206) and a relatively high number of visits (median: 8; IQR: 6–11) and several recurrent lapses/relapses (median: 5; IQR: 4–5). In RTF, the median time to first lapse of abstinence was 2 days (IQR: 2–9.5). This was followed by a relapse at a median time of 22 days (IQR: 9.5–64.5). The percentage of participants among RTF that were abstinent at one month, was 24%. STR similarly had frequent lapse/relapse alternations. PSR typically displayed a shorter follow-up time and a reduced number of visits. Nevertheless, cessation interventions usually triggered a substantial reduction of tobacco consumption in PSR by a median number of 5 cigarettes (IQR: 0–16).

## DISCUSSION

In this observational real-world study, we could identify four distinct smoking cessation phenotypes during assisted smoking cessation, which were both associated with participant’s baseline characteristics and chances of quitting success. As a novel approach in the field of smoking cessation, we further characterized the different phenotypes by using the personas methodology.

Previous studies were able to identify distinct patterns of quitting trajectories or smoking reductions^12-16,30^. These studies typically identified between three and five patterns most frequently described as quitters, reducers, persistent smokers and slow or quick returners. As an example, Bachmann et al.^12^ showed that besides the persistent smokers and fast quitters, two additional trajectories may exist, namely the late quitters and the returners. Their pattern detection was based on the analysis of self-reported information on the participant’s daily tobacco consumption within 29 days following the initial counseling visit. A variety of statistical approaches were used to identify quitting trajectories. Bachmann et al.^12^ used ecological momentary assessment to study relapse process, and latent class growth modeling to identify quitting trajectories. Mixed modeling approaches were used in other studies including those by Conklin et al.^13^ and Wong et al.^14^, while Hoeppner et al.^15^ used time-series typology procedures. Both experimental designs (follow-up time and frequency of smoking consumption monitoring) and analytical approaches, differed among studies. However, most studies agreed that smokers enrolled in smoking cessation programs exhibit quitting trajectories that are more diverse than previously assumed. These studies emphasized that daily observations allow detecting clear specific quitting patterns beyond the dichotomized abstinent versus smoker types, and opens the possibility for phenotype-based smoking cessation interventions in the transition phase.

The statistical approach of the current study is based on time-series clustering using PCoA. This methodology is both computationally efficient and provides attractive visualization tools to identify observations exhibiting similar patterns. We could identify four distinct transition phenotypes, which were clearly associated with baseline characteristics and outcomes. According to these findings, the accurate analysis of baseline characteristics and data from follow-up as well as phenotypization of the patients are useful for clinicians seeing new patients, since they give a chance to bring more personalized and ‘as needed’ strategies for treatment. These findings are also stressed by our current study. In our opinion, the participants who relapsed with intermittence (STR und RTF) deserve particular attention from the counselor. They were variably associated with relapse risk (for various reasons such as nicotine withdrawal symptoms, insufficient family support or missing motivation). Further, we could identify a cluster of promising patients who gradually stopped smoking and remained abstinent for a prolonged period. This may mean that especially at the beginning and because of the initial difficulties experienced during cessation attempts, some patients need more attention and more frequent assistance to maintain the motivation level. For these participants it seems easier to quit smoking gradually, with reduction of daily consumption and with more frequent support.

Most people are advised to quit smoking abruptly, the Cochrane analysis by Lindson et al.^31^ showed that reduction-to-quit interventions may be more favorable in some subgroups of smokers. Other studies showed that the number of visits is associated with greater chance of success during an assisted smoking quitting attempt^6^.

Some patients may also benefit from medication as an important step to lower nicotine dependence/tobacco consumption before attempting total smoking abstinence. With the current cluster analysis, we could identify possible predictors of successful quitting, which in turn can improve the outcome of a quit attempt. These factors may be either internal (predisposition/genetic, motivation level, self-confidence, psychological) or external (social, environmental) such as the use of NRT, support from family or family history of smoking. Previous clinical trials showed that older age, self-confidence in quitting, number of cigarettes smoked per day, number of previous quit attempts, lower levels of nicotine dependence, time to first cigarette after awakening, high-income and higher education level, were positive determinants of more successful smoking cessation^32-34^. The presence of these predictors should be examined prior to smoking cessation interventions, which may allow for a more individualized therapy. In contrast, our study could not identify a significantly different nicotine dependence within the four groups, whereas high motivational level and self-confidence were strong predictors of success. This may assume that psychological aspects are weighted more than actual nicotine dependence as a cause of lapse and relapse. These findings are comparable with results of West et al.^35^ who suggested that a sufficient motivational tension in the presence of adequate triggers (resulting in change of motivational state) and availability of treatment such as behavioral counseling may lead to successful smoking cessation, also without planning.

**Figure 4:**
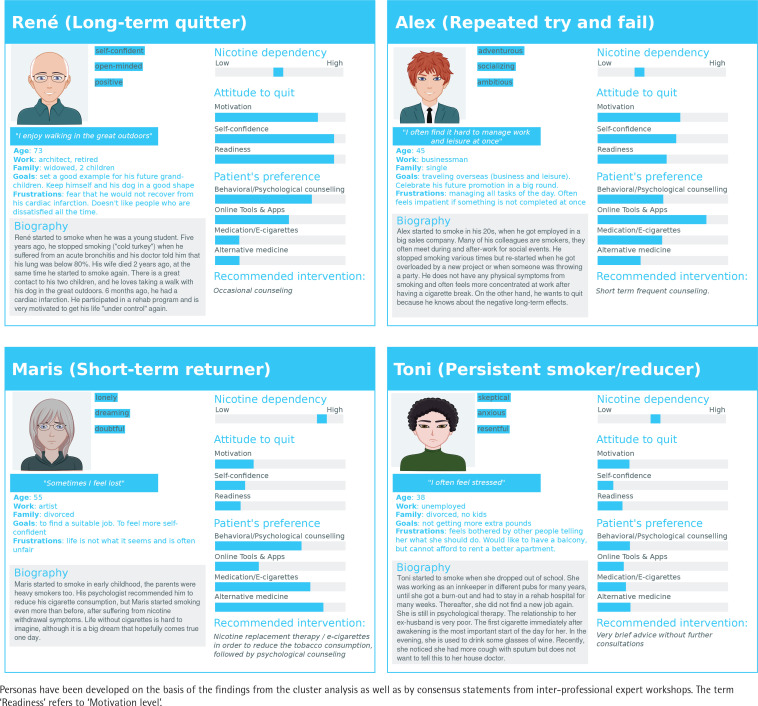
Cartoon of the 4 transition phenotypes using persona interpretations

All these assumptions have been integrated in our personas cartoons with the corresponding biography, motivational level and recommended intervention. The resulting four personas are shown in Figure 4. The characteristics were based on current and previous study results as well as on the high experience of our interprofessional smoking cessation team. Using the four personas, we were able to create a kind of physical and psychological profile of the patients as a useful tool for supporting the smoking cessation as well as a communication process with the participants. We focused mainly on internal and external factors that affect the quitting process and assessed the patients’ preferences regarding the intervention. We believe that most participants of smoking cessation programs will be able to identify and match with one of the four personas. The personas method can raise the awareness of the needs and behavior of the smokers. The phenotyping into these four personas may be done in most cases during the first consultation using baseline information as well as basic measurements such as FTND and evaluation of motivation level and self-confidence. The advantage of these management-relevant phenotypes is a high level of practical relevance as well as universality, accessibility and adaptability in smoking cessation interventions.

### Strengths and limitations

The limitations of our study include the retrospective nature of the study and the lack of real-time (daily) information on tobacco consumption. Because of the retrospective nature of our study, we do not have systematic quantitative data about the use of medications in each group. A future study would be required to investigate this aspect. Further limitations include the short follow-up time for many of the participants and that the study was conducted using data from a single, specialty center (smoking cessation clinic in Cantonal Hospital St. Gallen). Despite the fact that the data were collected a few years ago, they are still relevant as the patients’ baseline characteristics did not significantly change in the meantime.

The strength of our real-world retrospective study is its large sample size (n=843) and its duration of follow-up of up to 6 months compared to previous studies, which were typically limited to 1 month of observation. While most of the participants had a follow-up time of more than 6 months, to ensure the accuracy and quality of data, we decided to restrict data analysis to a follow-up time of 6 months – suitable for our focus on early tobacco consumption phenotype identification.

Further clinical and prospective studies using phenotypes/personas are needed to clearly underline our assumptions and identify and validate the most successful intervention strategy.

## CONCLUSIONS

Four meaningful and potentially targetable smoking cessation phenotypes were identified, which might open the possibility for phenotype-based smoking cessation interventions. Personalized and ‘as needed’ strategies involving intensive follow-up monitoring combined with individualized coaching have the potential to increase the success rate of smoking cessation interventions.

## Data Availability

The data supporting this research are available from the authors on reasonable request.
