# Supplementary material for: Identification of smoking cessation phenotypes as a basis for individualized counseling: An explorative real-world cohort study
Source: Tob Induc Dis. 2022 Sep 23;20:81. doi: 10.18332/tid/152546 (PMC9502004; doi:10.18332/tid/152546)
Supplement: Supplementary file 1 [file TID-20-81-s1.pdf]

**Table 1**: Transition phenotypes during assisted smoking cessation as identified by former studies [12-16].

| Reference | Sample size (n) | Follow-up-time (days) | Number and description of the phenotypes |
|---|---|---|---|
| Bachmann M, et al. 2012 [12] | 230 | 29 | **4:** Quitters; Late Quitters; Returners; Persistent smokers |
| Conklin CA, et al. 2005 [13] | 108 | 365 | **5:** Abstinent; Low-level users; Moderate users; Slow-returners; Quick-returners |
| Wong DC, et al. 2011 [14] | 402 | 180 | **3:** Reducers; Persistent smokers; Quitters |
| Hoeppner BB, et al. 2008 [15] | 57 | 40 | **3:** Increasing; Constant; Decreasing |
| Cofta-Woerpel L, et al. 2011 [16] | 300 | 28 | **3:** Abstainers; Early lapsers; Late lapsers |

1) Bachmann M, et al. 2012: Quitters (persistent abstinence over 29 days); Late Quitters (later onset of not smoking); Returners (individuals with increasing smoking over 29 days after the quit attempt); Persistent smokers (smoked over 29 days)
2) Conklin CA, et al. 2005: the study examined daily cigarette intake at several points during the 365 days following the participants`quit date. Five patterns of smoking resumption were identified: Abstinent (maintained abstinence over the 365 days). Among the participants who relapsed, four distinct trajectories emerges: Low-level users; Moderate users; Slow-returners; Quick-returners
3) Wong DC, et al. 2011: Reducers (participants who cut down their daily cigarette consumption rate to less than 40% of the original rate within the first month after baseline counseling and maintained the reduced daily cigarette consumption rate afterwards, Persistent smokers (participants who slightly reduced their daily cigarette consumption after receiving telephone counseling and continued their smoking behavior); Quitters (participants who reported a dramatic reduction in smoking consumption at day 7, and eventually stopped smoking)
4) Hoeppner BB, et al. 2008: the study examined daily smoking rates following the end of an intensive smoking reduction program over the course of 40 days: 3 smoking patterns were identified: Increasing; Constant (maintained their average smoking rate); Decreasin
5) Cofta-Woerpel L, et al. 2011: Abstainers (abstinent both at day 7 and 1 month); Early lapsers (participants who lapsed during the first post-cessation week); Late lapsers (participants who were abstinent during the first week but lapsed by 1 month)

**Supplementary Figure 1: A reliability of the four phenotypes identified by principal coordinates analysis using a bootstrap resampling strategy in order to visualize the confidence regions of PCoA solutions.**

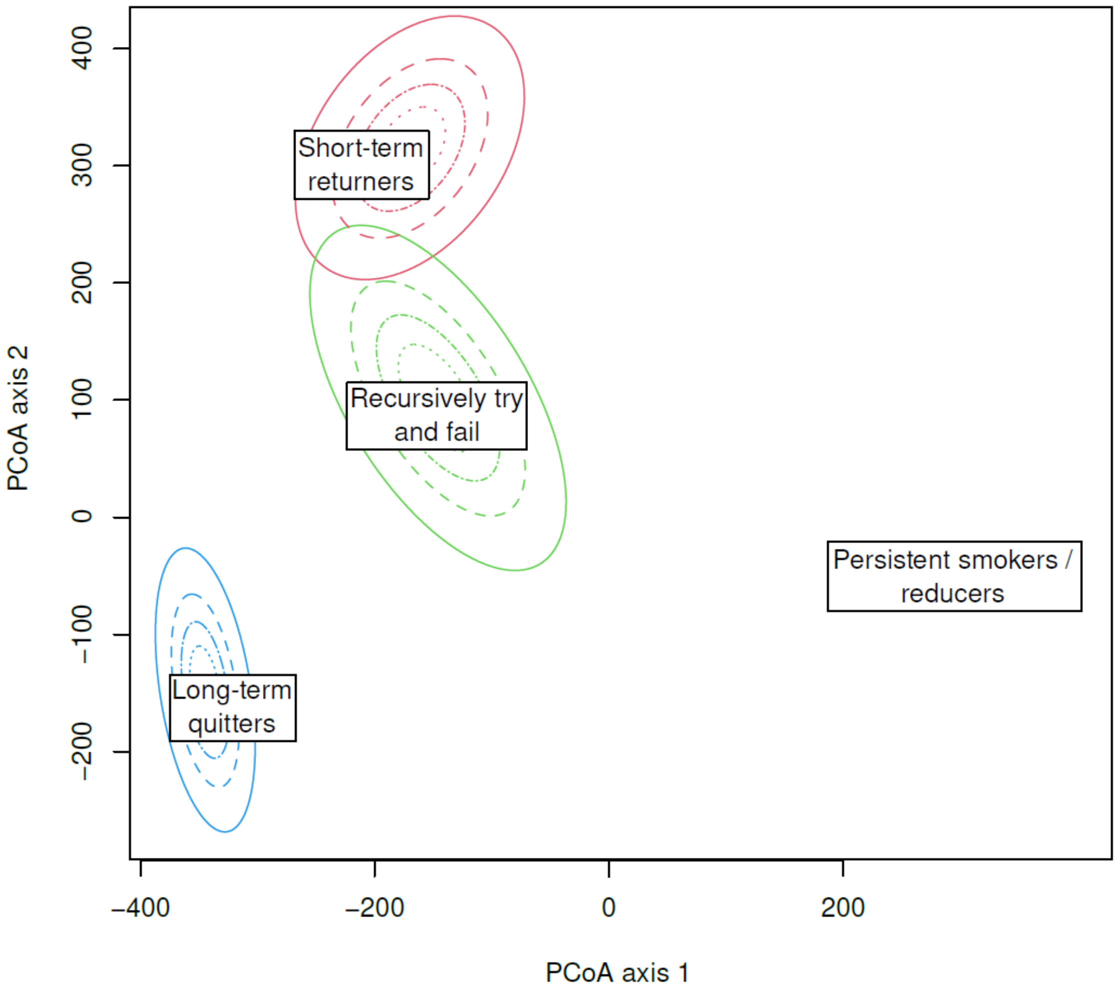


Bootstrap confidence regions are represented with ellipses with expected data fractions of data coverage of 95% (plain ellipses), 75% (dashed ellipses), 50% (dash-dotted ellipses) and 25% (dotted ellipses).

© 2022 Paciorkowski M. et al.
